# A Metadata-Based Approach to the Integration of Educational Resources in Ethnic Traditional Physical Education

**DOI:** 10.1155/2022/6505770

**Published:** 2022-09-12

**Authors:** Xiaodan Chen

**Affiliations:** Wushu School, Guangzhou Sport University, Guangzhou, Guangdong 510500, China

## Abstract

Ethnic traditional sports are a comprehensive interdisciplinary field that draws on and integrates theories and methods from related disciplines, particularly anthropology, to reveal the laws governing the emergence and development of ethnic traditional sports. The evolution of ethnic traditional sports has historically contributed to the construction of theoretical systems, the transmission of traditional culture, the formation of distinctive fitness techniques, the intensification of socioeconomic interests, and the development of cultural self-confidence. The majority of ethnic traditional sports teaching resources, however, are unstructured data including documents, images, animations, programs, tools, and videos. They rely heavily on ethnic traditional physical education teachers for instructional design, and there is no way to create a unified integration and sharing platform. From the preparation, supplementation, and description of resource metadata specifications to the clustering, integration, and cataloging of digital resources and the implementation of retrieval and service interfaces, this paper focuses on the effective integration of high-quality digital traditional ethnic sports resources in the process of network sharing.

## 1. Introduction

Numerous traditional ethnic sports are the treasures of Chinese cultural tradition. They have a lengthy history and should have an abundance of educational resources [1]. Nevertheless, the lack of systematization of educational resources has become the greatest weakness of teaching and learning in the process of teaching and research, causing great difficulties in the construction of curriculum systems, the exploration of educational theories, and the argumentation of literature [2]. Consider traditional martial arts as an illustration. The majority of the information on knife and stick techniques in martial arts that is widely available on the Internet and in print is simplified routines. There is a lack of systematization, and there is very little information about martial arts techniques that have been passed down orally from generation to generation among various martial arts schools. Consequently, educators and discipline builders cannot construct educational resources in an organized manner. In the past few thousand years, when information technology had not yet been developed, the succession and development of martial arts were frequently dispersed among noble families and schools, resulting in a paucity of complete and systematic texts [3]. In modern society, information technology provides favorable conditions, but the spirit of reverence and enthusiasm for martial arts has greatly diminished, and today's youth and parents of students place more emphasis on taekwondo, karate, or Western sports and martial arts routines, which results in a lack of information on traditional martial arts and absence of good channels for collecting, supplementing, and updating such information.

In addition, the following problems exist in the integration of college sports and national traditional sports resources:

1. Since the 21st century, with the acceleration of globalization, the globalization of Western economic sports has brought a great impact on the development of national traditional sports in China. Take the Olympic Games as an example [4]. At present, most of the Olympic events are Western athletic events. In the long run, people's concept of sports and competitive sports will be deeply rooted in the belief that the national traditional sports culture does not meet the characteristics of modern competitive sports. The misconception of our national traditional sports culture makes it more difficult for our national traditional sports to be carried out in colleges and universities.
2. Colleges and universities lack instructors who participate in traditional ethnic sports. There are a limited number of colleges and universities that specialize in ethnic traditional sports, and there is no effective training mechanism for the development of ethnic traditional sports instructors. Moreover, the number and caliber of teachers who specialize in ethnic traditional sports are inadequate.
3. The experience in carrying out ethnic traditional sports activities in colleges and universities is insufficient. For a long time, the content of physical education in colleges and universities has been influenced by competitive sports, and college students lack understanding of and interest in ethnic traditional sports activities. Meanwhile, due to the lack of experience, physical education teachers in colleges and universities are unable to exchange the equipment required for many ethnic traditional sports activities in the classroom or make it compatible. After school hours, colleges and universities still lack the awareness and experience of using sports competitions, sports festivals, and sports weeks to carry out some ethnic traditional sports activities.
4. The utilization rate of traditional ethnic sports resources in college sports is low. There are 56 ethnic groups in China. In the process of working and living, each ethnic group has created and formed folk traditional sports activities with various forms, rich contents, and distinctive ethnic characteristics and formed a whole ethnic cultural system with ethnic characteristics. Take Guangxi as an example. According to incomplete statistics, there are nearly 300 ethnic traditional sports in Guangxi alone. However, when choosing ethnic traditional sports items, college sports only focus on a few competition items and ignore many ethnic traditional sports items that integrate competitive entertainment, which leads to low utilization rate of ethnic traditional sports resources in college sports.

Information technology has permeated all facets of economic development and social life, and it has presented education with unprecedented opportunities and obstacles. Information technology has infused education with new vitality and vigor [5]. Not only does it provide innovative technical means and solutions and broaden access to high-quality resources, but it also injects new ideas and impetus for the sustainable development of education, promotes the reform of teaching methods, and facilitates the dual improvement of education quality and efficiency. Resource integration is the collection and organization of educational teaching resources and related literature to meet the needs of education and instruction. There is no clear and unified concept and method for resource integration at this time. There will be many difficulties in classifying, summarizing, and optimizing the use of resources due to the fragmented nature of the resource materials. This directly results in the inability to fully develop and utilize the basic materials available to people, as well as to disseminate and share them across the vast Internet landscape. It also reduces the efficiency and process of resource integration significantly.

This paper investigates an integration strategy for ethnic traditional sports resources based on metadata. Existing data can be fully utilized, and effective resource allocation and utilization can be attained through opening and sharing data. Metadata describing data resources can enable researchers to retrieve, access, and reuse data. This paper uses Data Catalog Vocabulary (DCAT) [6] as its theoretical foundation, analyzes DCAT metadata model, defines its classes and attributes, and maps and integrates the metadata information of multiple domestic portals by integrating data platform metadata. On the one hand, the paper inherits and develops the metadata theory and advances related theoretical knowledge; on the other hand, it combines the integration study of metadata with the actual of China's national traditional sports and provides theoretical information for future research on the data integration of educational resources. Moreover, this study has substantial practical significance for integrating metadata information of traditional ethnic sports resource platforms, based on the advanced experience of open data in other disciplines and at the management level. To guide the development of professional data platforms, we must first comprehend the educational resource data platforms' metadata standards and metadata practices. Second, the integration of information resources can guide the process of research affairs and further improve the efficiency of research and exploration. Third, the integration of metadata can aid users in discovering, utilizing, and sharing relevant data and datasets, as well as increasing the participation motivation of nonexperts.

## 2. Related Work

### 2.1. Ethnic Traditional Kinesiology Education Resources

In their paper titled “Research on Teaching Reform of Softball Based on Quality Online Educational Resources,” Liu et al. [7] analyzed the issues with softball's traditional teaching methods and proposed the application of quality online educational resources to the teaching reform of softball elective course. The study argued that the application of online educational resources in physical education teaching is an alternative to the traditional teaching method, as it improves the teaching effect, increases the quality and efficiency of instruction, and fosters students' independent learning and exercise skills. [8] In the article “Research on the Structure System of Chinese Ethnic Traditional Sports Discipline Education,” Yang et al. argued that the Chinese ethnic traditional sports discipline education system is facing new challenges, which necessitates playing the characteristic function of the discipline in discipline construction, meeting the current social demand for ethnic traditional sports discipline, enhancing the guiding ideology of ethnic traditional sports, and adapting to the requirements of contemporary society [9]. The article titled “Educational Resources Development and College Sports Reform” by Ma et al. begins with a summary of the relationship between teaching resources and college sports resources before separating teaching resources into teacher resources, equipment resources, and financial resources. Reform of college physical education cannot be separated from educational resources, and teacher resources are the foundation of teaching reform, improve sports equipment resources and the utilization rate of equipment resources, and improve utilization of educational resources. In addition, the development of college physical education resources is proposed to adhere to the principles of economy, sharing, and relevance.

In addition to macro optimization of various types of educational resources in higher education, specific studies on the educational resources of different disciplines should be conducted for the study of educational resource allocation [10]. The professional educational resources of ethnic traditional sports colleges and universities are both explicit and implicit resources of higher education. Explicit educational resources include teachers, teaching environment (teaching aids, venues, equipment, etc.), and study funds. Based on the characteristics of utilizing ethnic traditional sports, implicit educational resources can be separated into scientific research resources and curriculum resources.

Scientific research conditions that are conducive to the cultivation of graduate students in ethnic traditional sports include not only various forms of knowledge or information sharing, but also the integration of science and education, such as academic exchanges within or between research teams, laboratory sharing, and sharing of research platforms. Currently, the majority of ethnic traditional sports majors are scientific research teams led by instructors [11]. Through preliminary comprehension, their advantages are that the team has a clear research direction, with more mature research methods, and the supervisor has personal ability to lead graduate students to certain success. Similarly, their disadvantages consist of certain restrictions that may hinder the innovative growth of graduate students. Therefore, academic communication within research teams and communication between different collaborative research teams are necessary. Moreover, ethnic traditional sports science is a super-interdisciplinary field that can create synergy with the four secondary disciplines of other sports sciences, particularly in terms of empirical research. Empirical research resources are more abundant in other disciplines, so the sharing of research resources is also crucial for ethnic traditional sports graduate students. At the level of theoretical courses, experts and scholars of ethnic traditional sports in our school can offer theoretical courses or teach research experience to postgraduates, even retired old professors explained in person, or invite experts of ethnic traditional sports from other schools to deliver targeted lectures. In addition to the techniques and teaching tools mastered by school teachers, masters of martial arts or intangible cultural heritage from outside the school can be invited to teach technical courses to graduate students at the practical level. It is known that few colleges and universities have specifically invited experts, scholars, or martial arts masters from other colleges and universities to teach classes for postgraduates, and these course resources are unquestionably extremely valuable.

### 2.2. Metadata

In the era of big data, the importance of data has been raised to an unprecedented level. The abundance of information has also led to confusion in the information world. It is difficult for information users to find the useful information they need from the sea of information. This requires information publishers to improve information description methods, broaden information exchange channels, and reduce the difficulty of user data processing. Therefore, metadata was born. As information describing data, metadata can improve the accuracy of content description and enable users to judge whether open data is suitable for them [13].

#### 2.2.1. Concept and Classification of Metadata

Regarding metadata, the National Information Standards Organization [14] defines it from its own etymology; that is, metadata is data about information or data that is used to create, interpret, and share said content [15]. Metadata is structured information with abstract concepts that is mainly used to locate, describe, and interpret data resources and help users to retrieve, utilize, and share information faster and better. According to the different functions of metadata, NISO classifies it into four categories: descriptive metadata, administrative metadata, structural metadata, and markup language. See Table 1 for details.

#### 2.2.2. Metadata Mapping

To solve the interoperability problem between different metadata, metadata mapping is needed. Metadata mapping requires a specific transformation program to convert elements, syntax, and semantics of different metadata formats. For metadata mapping, a mapping table or mapping dictionary can be produced to show the mapping relationships between specific metadata in the form of a table. In order to develop a sound and scientific metadata mapping table, not only the transformation description of metadata but also the semantic mapping between elements is needed. The semantic mapping of elements should establish correspondence between elements according to the semantics, which is mainly divided into one-to-one relationship, one-to-many relationship, many-to-one relationship, and other relationships.

#### 2.2.3. Open Data Platform

Tao designed a quality assessment system and a quality assurance mechanism to address six issues with the quality of metadata in Chinese open data platforms [16]. Lang et al. investigated the current quality status of Chinese provincial open data platforms in four dimensions (data integrity, metadata, format and update, and data collection) and ten subordinate index systems and proposed targeted enhancements [17]. Meng et al. [18] conducted a comprehensive analysis of some domestic open data platforms based on the number of data applications, data topic classification, data development themes, and browsing and downloading functions and proposed deficiencies and countermeasures for application development. Using the Fuyang open education data platform as an example, Liao et al. [19] proposed suggestions and countermeasures to improve public satisfaction via a questionnaire survey.

#### 2.2.4. Research on the Construction of Metadata Standards for Open Education Data

In terms of metadata standard construction, Zhao Rongying took data.gov.uk as the research object and summarized the characteristics of metadata standards of UK data platforms in terms of metadata types, CKAN record formats, and geospatial metadata standards [20]. Yang et al. conducted a comparative analysis of Chinese and US dollar data schemes in terms of metadata formulation standards, metadata elements, and metadata formats and explained their similarities and differences [17].Wu Lin et al. conducted a comparative analysis of metadata standard policies related to open educational data in the United States, the United Kingdom, Australia, Canada, and the European Union and then compared the characteristics of these five countries in terms of file formats, metadata standard frameworks and classifications, DCAT synonym database support, and use of controlled synonym database [21].

There is a dearth of analytical research on open data platforms from the perspective of metadata integration; however, research on information resource integration in other fields can be consulted. In his research on the integration of educational information resources and library documentary resources, Li Pengyun developed integration concepts and system architecture based on an examination of the concept of library integration [22]. Wang Lancheng et al. combined top-down and bottom-up approaches to design a metadata scheme for archival socialized media information organization, integrate media information, and develop an integration system [23]. Zheng Lei analyzed the principal stakeholders of China's open education data platform and their interactions from an ecosystem perspective in order to promote the long-term growth of the entire open data system [24]. Jin Yuanbao provided ideas and countermeasures for the integration of information resources on educational websites in the era of big data [25].

## 3. Method

### 3.1. Metadata Standards

#### 3.1.1. Metadata Model Framework Analysis

**Figure 1:**
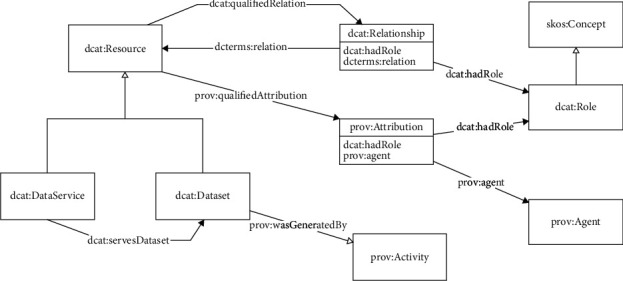
Metadata model.

DCAT stands for Data Catalog Vocabulary. DCAT builds the metadata model in the form of ontologies (RDF vocabularies) (as shown in Figure 1), which mainly includes top-level classes (datasets, data catalogs, catalog records, data resources, reusable class agents, conceptual schemas, and concepts defined by DCAT) and application classes (data services and data resources). DCAT reuses the Dublin Core metadata standard, the FOAF ontology, the SKOS ontology, and the W3C traceability ontology PROV-O [26].

#### 3.1.2. Main Classes and Relationships between Classes

DCAT specifies seven classes and their respective attributes, namely, dcat:catalog, dcat:dataset, dcat:resource, dcat:catalogrecord, dcat:dataservice, dcat:distribution, and foaf:agent. As shown in Figure 1, subclass relationships and partial relationships predominate. According to the number of outgoing and incoming relationships between classes, the DCAT model's primary classes and class relationships are analyzed.

As shown in Figure 1, the data catalog class (dcat:catalog) is the class in the DCAT metadata model with the most outbound and inbound relationships. This class descends from the dataset class. This subclass has six outlink relationships to the dataset (subclass relationships and dataset relationships), data resources, data services, catalog records, and FOAF proxy classes, as well as one proxy relationship to itself. The dataset class and the data services class are included in the data catalog class. It is a concrete example of open government data portals such as catalog data.gov and bjdata.gov.cn. The dataset class (dcat:dataset) represents a dataset published and managed by a single agent, which can be accessed or downloaded via one or more serialization or format [27]. This class serves as the parent and has no linked-out properties. These properties are organized into distinct subclasses. This class is linked to the data catalog (2), data publishing, and data services via four relationships. On the data catalog website, an abundance of datasets will be made available. The definition of the data resource class (dcat:resource) is a resource published or managed by a single agent. It is an extension point for any resource catalog type. A data resource class is a model class with an outer link property relationship that exceeds the second level. Four outlink relationships connect SKO's concept classes, FOAF's agent classes, and related classes. The final outlink refers to the property dct:conformsto.

The data quality vocabulary (DQV) and the traceability ontology PROV-O are used to describe information that is more complete and is of higher quality. This class contains three relations derived from the data catalog, catalog records, and relations. The remaining three classes have comparatively few external and internal linkages. The catalog record class (dcat:catalogrecord) represents the number of records of a dataset in a catalog; the data service class (dcat:dataservice) is instantiated by APIs and so on; and the data distribution class (dcat:distribution) represents the access or download method for a dataset. The DCAT model makes reference to a significant external class: the FOAF proxy class, which is the parent of human and institutional classes in other models and is used to represent the publisher or creator of data catalogs and data resources.

#### 3.1.3. Element Composition

DCAT defines different categories of metadata, as shown in Table 2. The data catalog contains all elements of the dataset and data directory, and the data catalog, dataset, and data service contain elements related to data resources.

### 3.2. Metadata Integration System Architecture

**Figure 2:**
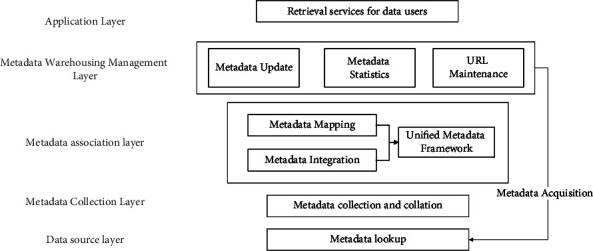
Metadata integration system architecture diagram.

This study proposes an education resource data integration system based on metadata. Its primary functions include monitoring and analyzing the metadata information content of data platforms in real time, filtering and automatically clustering and classifying the collected metadata information, aggregating metadata information from multiple platforms, achieving unified data information display and navigation, providing metadata query and entity data query functions, and presenting visual information presentation. Initial construction of the metadata integration system of the national traditional sports resource data platform is based on the principle of metadata storage. As depicted in Figure 2, in order to integrate our framework, we must first obtain data resources and dataset information from various open data platforms, classify and organize the metadata, divide the levels based on the teaching content, perform metadata mapping and metadata integration separately, and establish a unified metadata framework. Through metadata update, metadata statistics, and URL maintenance, this framework enables the retrieval service for data users.

### 3.3. Metadata Mapping

#### 3.3.1. Data Resources

We selected a university, an offline community, and an online education platform for data mapping, as shown in Table 3. In terms of metadata items of data resources, except for the metadata items of “release date,” “update date,” and “file size,” the the US and DCAT synonym lists basically map the metadata items of the two to each other. For China, the metadata items of data resources are relatively lacking. All three platforms have metadata items, such as name, description, release date, update date, and file format. The university and offline community have metadata items for “file size” and “link address.” The online education platform has a metadata item for “file download URL.”

#### 3.3.2. Dataset Metadata Mapping

In mapping the dataset metadata (Table 4), it can be seen that for each metadata information item of DCAT, the US has corresponding metadata with the same metadata description method. The three selected data platforms have different metadata descriptions for the datasets. The descriptions for universities and offline communities are basically the same. Only the two open government data platforms have different “description” metadata items. The university is “data description” and the offline community is “summary.” The online education platforms have less content in their datasets. For example, there is no “link address,” “language type,” “spatial extent,” “temporal extent,” and the descriptions of some metadata are different from those of Shenzhen and Guiyang. The description of “name” is “resource description,” as well as “label” and “keyword.” “Contact” and “Provider Address,” “First Release Date,” and “Release Date,” etc. are all things to look into.

### 3.4. Metadata Integration

Based on the structural analysis of DCAT synonym table and its extended application analysis in the US, after mapping the metadata information of each platform to DCAT and the US metadata scheme, we try to construct domestic metadata based on ontology semantics to complete the opening and integration of different sources and structures. Metadata integration is to classify metadata in the same data domain, map metadata with identical or the same meaning but different element names into a unified metadata framework, represent metadata in the same data domain in a unified way, and reduce the differences between metadata as much as possible while retaining the same category of data information. The important purpose of metadata integration of open government data platforms is to collect data information of multiple categories and carriers to form a rich large-scale resource database, which enables government agencies to comprehensively manage and share services for all data resources. At present, the metadata information of each platform has not been unified, so the data integration in this study is mainly based on the current metadata status of each platform. Most of the data catalogs of the national traditional sports resource data platform are classified according to organizations and subject sets. To realize the integration of metadata, it is necessary to divide existing resources by resource type from bottom to top according to the resource type.

We use the basic logical framework of the integrated system in Figure 2 to manage the presence of each functional module in the system and the related management operations between the modules. The application layer is designed to receive the input pattern of the system of intelligent information and to give the integrated results back to the operator. In the second layer, the business logic layer, the business logic components are set up according to the actual tasks of the management function modules [27]. The relevant index for deploying each logical component is set by the following equation:

(1) $$\begin{array}{c} K_{i}=\frac{\left(x_{i}/x_{j}\right)}{\left(X_{i}/X_{j}\right)}, \end{array}$$

where *K*_*i*_ denotes spatial location entropy; *x*_*i*_ and *x*_*j*_ represent Class I and Class J library intelligence, respectively; and *X*_*i*_ and *X*_*j*_ represent different types of libraries and information sources, respectively. The third layer is the support layer, which mainly provides data and functional support for the information integration of various logical components. In terms of data, the basic structure of distributed database is adopted to unify the management of information sharing operations and support the metadata service, item import and export service, and web user service of DSPACE; it provides business logic components in terms of function support, information retrieval, query, and classification download functions, and realizes the management of the entire library information. Finally, the data storage layer is designed to handle library intelligence literature data and information data [28].

## 4. Experimental Setup and Analysis

### 4.1. Experiment Preparation

Select the experimental equipment, create a simulated test environment, simulate the content of the client-initiated request, and evaluate the integrated system's stress tolerance in the face of the massive amount of book information. Send HTTPS requests while testing the application of the integrated system using Apache server software. The examination was divided into five phases, with the fields utilized in each phase listed in Table 5. The objectives of this experiment were to determine the maximum TPS that could be achieved with a single machine deployment and whether or not the TPS could reach the expected value of 300. After completing the preceding preparations, 15-minute trial runs were conducted to ensure that the system's hardware was functioning properly. If no problems were discovered, the experiment could begin.

### 4.2. Concurrent Performance Testing

In the four concurrent performance tests, one process is started, and 50, 100, 300, and 500 threads are started for a single process. The results are calculated and exported as shown in Table 6.

The integrated system was stressed under 100 vu, 500 vu, 1000 vu, and 2000 vu load conditions. According to the test results presented in Table 4, the user successfully loaded at 500/s under all four test conditions. Users descended at a rate of 500 seats per second under the 5-minute test time. The response times for the first three groups were below the test standard value of 3 seconds, whereas the response time for 2000 vu was greater than 3 seconds. The data indicate that the error rate of the integrated system is within an acceptable range when there are fewer than one thousand concurrent users.

### 4.3. Classification Performance Test

The quality of integration of library information resources of different systems was compared with the system as the experimental group and the integrated system under the traditional design as the control group. The integrated quality evaluation index is used as the basis for analyzing the system performance, and the accuracy measurement method is used to calculate the root mean square error of the prediction result accuracy. The calculation results of the two parameter values can be directly derived by the following equation:

(2) $$\begin{array}{c} \left\{\begin{array}{c} \begin{array}{ccc} \text{MAE}\left(\mu \right) & = & \left(\frac{1}{\left|N\right|}\right)\underset{r_{i}\in R}{\sum }\left|r_{i}-r^{\prime }\right|, \end{array}\\ \begin{array}{ccc} \text{RMSE}\left(\mu \right) & = & \sqrt{\left(1/\left|N\right|\right)\underset{r_{i}\in R}{\sum }{\left(r_{i}-r^{\prime }\right)}^{2}}, \end{array} \end{array}\right. \end{array}$$

where MAE (*μ*) denotes the mean absolute error; RMSE (*μ*) denotes the root mean square error of the accuracy of the prediction result; *N* denotes the number of items rated by users; *RI* represents the actual rating of the system by users; *R′I* denotes the prediction score of the system by users; and *R* denotes the score set. The smaller the calculated values of MAE (*μ*) and RMSE (*μ*), the higher the degree of matching between library intelligence information and integrated classification of the resource integration system. Using the above calculation as the evaluation basis for the test results, any five datasets were selected in the MovieLens 100K dataset, and the dataset was trained and tested in the ratio of 3 : 1. The performance evaluation results of different resource integration systems are shown in Table 7.

According to the above calculation results, the MAE and RMSE values of the designed system are low, indicating that the library intelligence information integrated by the system in this study is closer to the classification results. Further, we conducted hypothesis testing and performed *t*-test on the data of the two methods on the two indicators, and the obtained *P* values were all less than 0.05, indicating that the method in this paper is statistically significantly better than the traditional method.

## 5. Conclusion

In the context of the Internet and education, with the acceleration of the deep integration of information technology and education teaching, the teaching design of traditional ethnic physical education teachers is frequently singular and unsystematic and cannot form a unified platform for integration and sharing. An approach based on metadata is proposed for the integration of traditional ethnic sports educational resources in response to these diverse educational resources. The metadata model is one of the study's central contributions. By analyzing the current situation of metadata of each ethnic traditional sports teaching resource platform and the current situation of integrated resources of each district and school, a common template of metadata is designed, and a creative three-level structure of metadata core, extension, and supplement is proposed, making the model highly scalable and adaptable and providing a foundation for the establishment and exchange of a database. With the assistance of this model, a more comprehensive set of digital education resource integration and fusion engineering methods is established, which provides the best solution for teachers of ethnic traditional sports in the resource application process, maximizes the support of teachers' work efficiency, and promotes the improvement of curriculum teaching level.

In the future, we will try to integrate the method of this paper into the teaching of other subjects, such as mathematics and physical education. Further, in the future we will try to integrate a more advanced metadata-based approach to integrate educational resources.

## Figures and Tables

**Table 1:** Classification of metadata.

Metadata categories		Usage examples
Descriptive metadata		Title, author, subject, type, date of publication
Administrative metadata	Technical metadata	File type, file size, creation date/time
	Preservation metadata	Validation, data update and migration
	Copyright metadata	Copyright status, license terms, copyright holder
Structural metadata		Table of contents, chapter and paragraph details
Markup language		Paragraphs, headings, tables, names, dates

**Table 2:** Metadata model element composition.

Metadata category	Element composition
Catalog	foaf:homepage, dcat:themeTaxonomy, dct:hasPart, dcat:catalog, dcat:record

Resource	dct:accessrights, dct:conformsto, dct:keyword/tag, dct:license, dct:contactpoint, dct:creator, dct:description, dct:relation, odrl:haspolicy, dct:identifier, dct:isreferencedby, dct:landingpage, dct:type/genre, dct:rights, dct:qualifiedrelation, dct:publisher, dct:releasedate, dct:theme/category, dct:title, dct:resourcelanguage, dct:update/modificationdate, dct:qualified attribution

Dataset	dcat:distribution, dcat:frequency, dcat:spatial/geographiccoverage, dcat:spatialresolution, dcat:temporalcoverage, dcat:temporalresolution, prov:wasgeneratedby

Catalog record	dct:conformsto, dct:description, dct:listingdate, foaf:primarytopic, dct:title, dct: update/modification date

Distribution	dct:accessrights, dct:accessURL, dct:accessservice, dct:bytesize, dct:compressionformat, dct:conformsto, dct:description, dct:downloadURL, dct:format, dct:haspolicy, dct:license, dct:mediatype, dct:packagingformat, dct:releasedate, dct:rights, dct:spatialresolution, dct:temporalresolution, dct:title, dct:update/modification date

Data service	dcat:endpointdescription, dcat:endpointURL, dcat:license, accessrights, dcat:serves dataset,

**Table 3:** Data resource metadata mapping.

DCAT	University	Offline societies	Online platform
dct:title	Document name	Name	Name
dct:description	Abstract	Abstract	Abstract
dct:issued	First release date	Release date	Release date
dct:modified	Update date	Update date	Update date
dcat:byteSize	File size	File size	File size
dct:format	Resource format	Resource format	Resource format
dcat:accessURL	Online resource link	Online resource link	Online resource link
dcat:downloadURL	DownloadURL	Null	Null
dct:conformsTo	ConformsTo	Null	Null
dcat:mediaType	MediaType	Null	Null

**Table 4:** Metadata mapping of datasets.

DCAT	University	Offline societies	Online platform
dct:title	Resource name	Name	Name
dct:description	Abstract	Summary data	Abstract
dcat:keyword	Tag	Keyword	Keyword
dct:publisher	Provider data	Provider data	Provider data
dcat:contactPoint	Contact information	Provider's address	Provider's address
dct:issued	First release date	Release date	Release date
dct:modified	Update date	Update date	Update date
dct:accrualPeriodicity	Update cycle	Update frequency	Update frequency
dct:license	Open properties	Open mode	Open mode
dcat:theme	Data domain	Data domain	Data domain
dct:landingpage	Null	Resources online	Resources online
dct:language	Null	Language	Language
dct:spatial	Null	Spatial scope	Spatial scope
dct:temporal	Null	Time range	Time range
dct:identifier	Null	Identifier	Identifier

**Table 5:** Description of the fields used.

Index	Field	Remarks
1	Label	Name of the element attribute of the test tool
2	Samples	Number of requests
3	Average	Average response time
4	Median	50% response time of users
5	Min	Minimum response time
6	Max	Maximum response time
7	Error (%)	Error request rate
8	Throughput	Number of requests completed per second
9	Received (KB/s)	Amount of data received per second

**Table 6:** Result analysis table.

Test content	50	100	300	500
Total number of strokes	225 015	216 107	198 198	98 579
JMeter number of error strokes	381	1949	11025	32661
90% response time of users (ms)	397	939	2799	10196
Throughput (s)	704	608	550	300
Average response time (ms)	263	688	1503	3021

**Table 7:** Performance comparison of different systems.

Test data	Our system		Traditional integrated system	
	MAE	RMSE	MAE	RMSE
Data1	0.6729	0.9324	0.8679	1.5732
Data2	0.6015	0.8891	0.9062	1.6830
Data3	0.6094	0.8237	0.9041	1.7203
Data4	0.6648	0.8331	0.9187	1.7182
Data5	0.6831	0.8212	0.8792	1.6934

## Data Availability

The data used to support the findings of this study are available from the author upon request.
